# Long-term efficacy and safety of insulin detemir compared to Neutral Protamine Hagedorn insulin in patients with Type 1 diabetes using a treat-to-target basal–bolus regimen with insulin aspart at meals: a 2-year, randomized, controlled trial

**DOI:** 10.1111/j.1464-5491.2007.02407.x

**Published:** 2008-04-01

**Authors:** P C Bartley, M Bogoev, J Larsen, A Philotheou

**Affiliations:** * Department of Medicine, University of Queensland Australia; † Clinic of Endocrinology and Metabolic Disorders Skojpe, Macedonia; ‡ Novo Nordisk A/S Bagsvaerd, Denmark; § Endocrine Diabetes Unit, University of Cape Town South Africa

**Keywords:** insulin analogues, glycaemic control, hypoglycaemia, Type 1 diabetes, HbA_1c_

## Abstract

**Aims:**

This 24-month, multi-national, open-label, parallel group trial investigated the long-term efficacy and safety of insulin detemir and Neutral Protamine Hagedorn insulin in combination with mealtime insulin aspart in patients with Type 1 diabetes using a treat-to-target concept.

**Methods:**

Patients were randomized 2 : 1 to detemir (*n* = 331) or NPH (*n* = 166) groups. Basal insulin was initiated once daily (evening) and titrated individually based on self-measured plasma glucose (PG) levels, aiming for pre-breakfast and pre-dinner targets ≤ 6.0 mmol/l. A second basal morning dose could be added according to pre-defined criteria.

**Results:**

After 24 months, superiority of glycated haemoglobin (HbA_1c_) was achieved with detemir compared to NPH (detemir 7.36%, NPH 7.58%, mean difference −0.22% points) [95% confidence interval (CI) −0.41 to −0.03%], with reductions of 0.94% and 0.72% points, respectively. Fasting PG (FPG_lab_) was also lower with detemir (detemir 8.35 mmol/l, NPH 9.43 mmol/l; *P* = 0.019). Twenty-two per cent of patients treated with detemir reached an HbA_1c_ ≤ 7.0% in the absence of confirmed hypoglycaemia during the last month of treatment vs. 13% on NPH (*P* = 0.019). Risk of major and nocturnal hypoglycaemia was 69% and 46% lower with detemir than with NPH (*P* < 0.001), respectively; patients treated with detemir gained less weight (detemir 1.7 kg, NPH 2.7 kg; *P* = 0.024). The overall safety profile was similar in the two groups and treatment with detemir did not result in any unexpected findings.

**Conclusions:**

Long-term treatment with the insulin analogues detemir + aspart was superior to NPH + aspart in reducing HbA_1c_, with added benefits of less major and nocturnal hypoglycaemia and less weight gain.

Diabet. Med. 25, 442–449 (2008)

## Introduction

Studies such as the DCCT have shown that intensive insulin therapy can improve glycaemic control and thereby reduce the risk of micro- and macrovascular complications faced by patients with Type 1 diabetes [1,2]. However, as glycated haemoglobin (HbA_1c_) is reduced, the risks of hypoglycaemia [3] and weight gain increase [4] and become the main barriers for obtaining good glycaemic control [5–7]. These adverse effects may be more pronounced with conventional insulin products, which are characterized by a high degree of variation in absorption and pharmacodynamic profiles that are not well matched with physiological insulin requirements [8–10].

During the last decade, both rapid-acting and basal insulin analogues with more physiological and predictable action profiles have been developed. These are generally at least as effective in reducing HbA_1c_ as conventional human insulin, and are associated with benefits including reductions in post-prandial plasma glucose, lower risk of hypoglycaemia and less weight gain [7,11]. However, to date, many of the trials reported with these analogues have been of too limited duration to establish long-term efficacy and safety or the applied glycaemic targets have been inadequate to demonstrate the full potential of such analogue regimens.

Detemir is a long-acting basal soluble acylated analogue of human insulin with a protracted action profile because of hexamer stabilization at the injection site and buffering of insulin concentrations via albumin binding in the blood [12]. Clinical trials of ≤ 1 year duration have shown that detemir is associated with comparable HbA_1c_, less variability in fasting plasma glucose (FPG), less nocturnal hypoglycaemia and less weight gain compared to intermediate-acting NPH [13–16].

The current trial is the first to investigate the long-term (24 months) efficacy and safety of a basal–bolus regimen with detemir or NPH in combination with aspart at meals in patients with Type 1 diabetes using a treat-to-target concept.

## Subjects and methods

### Design

The trial was performed at 33 investigational sites in 10 countries worldwide. Patients were randomized to detemir or NPH in a 2 : 1 ratio using a telephone randomization system. Because detemir and NPH are visually distinguishable and patients were to self-administer insulin throughout the trial, an open-labelled design was used. The trial included 13 scheduled visits and an extensive number of telephone contacts to ensure close contact between participants and investigators.

### Patients

Patients [≥ 18 years, with an HbA_1c_ ≤ 11.0% and body mass index (BMI) ≤ 35.0 kg/m^2^] with a history of Type 1 diabetes ≥ 1 year treated on a basal–bolus insulin regimen for ≥ 3 months and able and willing to self-measure plasma glucose were enrolled. Individuals with proliferative retinopathy or maculopathy, other significant medical disorders, recurrent major hypoglycaemia, allergy to insulin and pregnant or breast-feeding women were excluded. The trial was conducted in accordance with Good Clinical Practice [17] and the Declaration of Helsinki [18] and was approved by the local ethics committees. Prior to trial entry, all participants gave written informed consent.

### Titration and treatment

All patients started on a once-daily basal insulin regimen and administered either detemir (Levemir®, Novo Nordisk A/S, Bagsvaerd, Denmark 100 U/ml) or NPH (Insulatard®, Novo Nordisk A/S, 100 IU/ml) at any time during the evening. Aspart (NovoRapid®, Novo Nordisk A/S, 100 U/ml) was injected immediately before each main meal. Basal insulin was administered in the thigh and aspart in the abdomen. Patients transferred from a once-daily basal insulin regimen started treatment with detemir or NPH at an identical number of units, while those transferred from a twice-daily regimen initiated treatment at 70% of the previous total daily basal insulin dose. Basal insulin was titrated individually throughout the trial aiming for a PG target ≤ 6.0 mmol/l before breakfast and dinner. Participants were instructed in the use and calibration of blood glucose meters and were asked to measure PG pre-breakfast and pre-dinner on three consecutive days prior to each contact. Based on the average of these measurements, the investigators titrated the basal insulin dose according to a simple algorithm (Table 1). If the FPG target was achieved while pre-dinner PG values remained above target, the basal evening dose could be increased as long as nocturnal hypoglycaemia did not occur. A second basal insulin dose could be added in the morning if the pre-dinner PG target was not achieved with use of the algorithm and after optimization of bolus insulin. The basal morning dose was initiated at 4 U and titrated according to the same algorithm as used for the evening dose (Table 1). Aspart was titrated according to local practice to achieve a post-prandial PG level ≤ 9.0 mmol/l. A central surveillance committee reviewed the PG concentrations and the prescribed basal insulin doses throughout the trial and contacted the investigator in case of recurrent deviations from the algorithm or in case of missing data.

**Table 1:** Algorithm used for titration of the basal insulin dose

FPG or pre-evening meal PG value (based on average SMPG values)	Adjustment of insulin dose (U)
> 15 mmol/l	+6 U
10.1–15.0 mmol/l	+4 U
6.1–10.0 mmol/l	+2 U
≤ 6.0 mmol/l	No adjustment
*If one SMPG measurement:*	
3.1–4.0 mmol/l	−2 U
< 3.1 mmol/l	−4 U

FPG, fasting plasma glucose; SMPG, self-monitored plasma glucose; U, units.

During the first 12 weeks, patients were in weekly contact with the investigator or research team (by phone, fax or e-mail). Thereafter, contact was made at least every month.

### Antibody assessment

Blood samples for analysis of insulin antibodies were drawn at randomization and after 64 and 105 weeks of treatment. To avoid interference from detemir in the blood during insulin antibody determination, patients treated with detemir were switched to NPH 4–8 days prior to blood sampling for antibody analyses at these visits. For safety reasons, the NPH dose was reduced by 20% during this period and after blood sampling the patient was transferred back to detemir at the same dose as before the switch.

### Analytical methods

Blood samples for analysis of HbA_1c_ and FPG_lab_ were drawn approximately every 3 months. HbA_1c_ was determined by HPLC (Bio-Rad Variant, Bio-Rad Laboratories GmbH, Munich, Germany), (reference range of assay: 4.3–6.1%). FPG_lab_ was analysed by a hexokinase method (Gluco-quant®; Roche Diagnostics GmbH, Mannheim, Germany). Standard analyses of haematology, biochemistry and lipids were performed by Laboratorium für Klinische Forschung, Raisdorf, Germany. SMPG was measured using a glucose meter (Medisense Precision Xtra™ or Optimum Plus™; Abbott Diabetes Care, Delkenheim, Germany). Use of test strips calibrated to PG values ensured that capillary blood concentrations were displayed as PG values. Body weight was measured at all scheduled visits. Hip/waist ratio was calculated as the mean of three measurements and skin-fold thickness was measured using callipers. All SMPG values < 3.1 mmol/l as well as signs and symptoms of hypoglycaemia were recorded in the patients’ diaries and included in the analysis of hypoglycaemia. Insulin antibodies (detemir-specific, aspart-specific and antibodies cross-reacting between detemir and aspart) were analysed by MDS Pharma Services, Fehraltorf, Switzerland using a subtraction RIA technique [19] modified for determination of detemir antibodies. All blood samples were obtained in the morning before administration of insulin. ECG and fundoscopy/fundus photography, physical examination and vital signs were evaluated at randomization and after 1 and 2 years of treatment.

### Statistical analyses

The trial was designed as a non-inferiority trial using a 2 : 1 randomization. The primary end-point, HbA_1c_ after 24 months, was tested for non-inferiority using a two-sided test at a 5% significance level by applying a closed testing procedure [20]. A total of 489 patients were needed to obtain 245 evaluable patients on detemir and 123 on NPH to detect a clinically relevant difference of 0.4% in HbA_1c_ with a power of 85%, assuming a standard deviation (sd) for HbA_1c_ of 1.2 and an expected drop-out rate of 25%. HbA_1c_ was analysed by an ANCOVA model with treatment and country as fixed effects and baseline HbA_1c_ value as a covariate. FPG_lab_, change in weight, hip/waist ratio, skin-fold thickness and leptin concentrations were analysed after 24 months using similar models with corresponding baseline values as covariates. The percentage of patients who reached HbA_1c_ ≤ 7.0% at the end of the trial without symptomatic hypoglycaemia with a PG < 4.0 mmol/l or any single PG value < 3.1 mmol/l during the last month of treatment was analysed using Fisher's exact test. Within-patient variation (sd), based on the average of all available SMPG pre-breakfast and pre-dinner measurements during the last week of treatment, was analysed using a mixed model. Nine-point SMPG profiles, recorded during the last week of treatment, were analysed using a repeated measures model.

Hypoglycaemic episodes were classified as major if assistance from another person was required, as minor if PG < 3.1 mmol/l and the individual dealt with the episode him/herself, and as symptoms only if episodes were not confirmed by a PG measurement and no assistance was required. Relative risk of nocturnal (23:00 to 06:00 h) hypoglycaemic episodes was tested separately. Changes in insulin antibodies were based on analyses of blood samples obtained at weeks 64 and 105. The statistical ANCOVA model included country and time as fixed effects with total basal insulin dose and HbA_1c_ at end of trial and baseline antibody levels as covariates.

## Results

### Patient disposition

**FIGURE 1:**
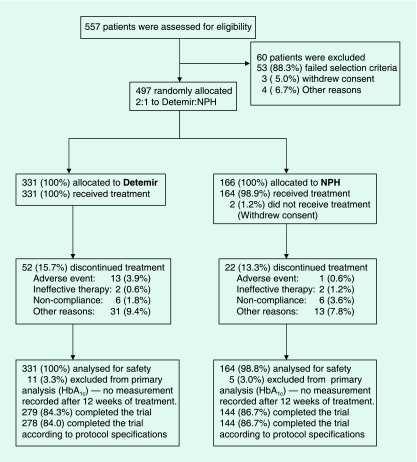
Patient disposition.

Of the 497 patients randomized, 495 were exposed and approximately 85% completed the trial (Fig. 1). A slightly higher proportion of patients on detemir withdrew because of adverse events, while withdrawal because of non-compliance was more common in the NPH group. A fairly large proportion of patients in both arms withdrew for ‘other’ unspecified reasons. With the exception of one patient treated with detemir, who withdrew because of concerns of hypoglycaemia, these were all non-related to trial products.

Patient characteristics were similar between the two groups (Table 2). This was also the case with respect to diabetic complications and concomitant illnesses (not shown). Approximately 60% of patients in both groups were on a once-daily basal insulin regimen prior to the trial and around 90% of all patients were treated with NPH at baseline. Mean daily insulin doses were similar between the two groups (Table 2).

**Table 2:** Baseline characteristics

Patients exposed to treatment, *n* (%)	Detemir 331 (100.0%)	NPH 164 (100.0%)	All 495 (100.0%)
Sex			
Female	147 (44.4)	77 (47.0)	224 (45.3)
Male	184 (55.6)	87 (53.0)	271 (54.7)
Ethnic origin			
White	244 (73.7)	129 (78.7)	373 (75.4)
Black	3 (0.9)	1 (0.6)	4 (0.8)
Asian/Pacific Islander	66 (19.9)	32 (19.5)	98 (19.8)
Other	18 (5.4)	2 (1.2)	20 (4.0)
Diabetes duration (years)	12.7 (1.0–50.4)	13.5 (1.1–49.4)	13.0 (1.0–50.4)
Age (years)	35 (18–75)	35 (18–70)	35 (18–75)
BMI (kg/m^2^)	24.7 (15.4–34.6)	24.7 (16.9–34.7)	24.7 (15.4–34.7)
Weight (kg)*	71.2 (39.5–128.4)	70.9 (33.6–119.0)	71.1 (33.6–128.4)
HbA_1c_ (%)*	8.3 (5.0–11.6)	8.4 (5.3–11.4)	8.3 (5.0–11.6)
FPG (mmol/l)*	11.4 (0.7–33.9)	11.7 (2.8–30.4)	11.5 (0.7–33.9)
Pre-trial insulin regimen			
1 basal + 3 bolus	194 (58.6)	100 (61.0)	294 (59.4)
2 basal + 3 bolus	88 (26.6)	42 (25.6)	130 (26.3)
Pre-trial daily insulin dose			
Basal insulin (IU/kg)	0.37 (0.04–1.10)	0.36 (0.06–1.24)	0.37 (0.04–1.24)
Meal-time insulin (U/kg)	0.46 (0.02–1.67)	0.45 (0.03–1.29)	0.46 (0.02–1.67)

Values are *n* (%) or mean (range).

NPH, Neutral Protamine Hagedorn; BMI, body mass index; HbA_1c_, glycated haemoglobin; FPG, fasting plasma glucose.

* HbA_1c_, FPG and weight recorded at or before randomization.

### Glycaemic control

**FIGURE 2:**
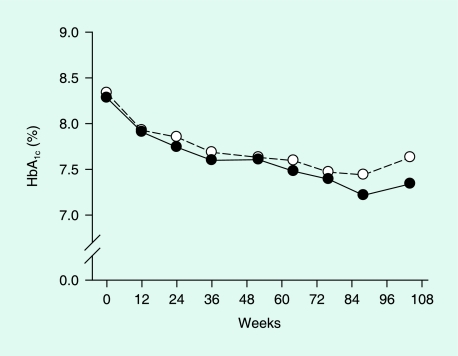
Change in mean glycated haemoglobin (HbA_1c_) over time. Detemir, black circles; Neutral Protamine Hagedorn, white circles.

HbA_1c_ decreased by 0.94% with detemir and by 0.72% with NPH during the trial; glycaemic control was superior with detemir after 24 months, with a mean difference (detemir—NPH) of −0.22% points (Table 3). The difference in HbA_1c_ between the two treatment groups was most pronounced during the last 6 months of the trial (Fig. 2). FPG_lab_ also decreased to a larger extent with detemir than with NPH, with reductions of 3.01 mmol/l vs. 1.93 mmol/l (*P* = 0.019) (Table 3). Within-patient variation in self-measured FPG was lower with detemir than with NPH (sd 2.18 mmol/l vs. 2.52 mmol/l; *P* < 0.001), while no statistically significant difference was found in pre-evening meal PG variation (sd 2.50 mmol/l vs. 2.46 mmol/l; *P* = NS). Self-measured nine-point PG concentrations were generally reduced in both groups at all times of the day, but the shape of these profiles could not be considered parallel after 24 months of treatment (*P* = 0.004). The main differences between treatments were related to higher mean PG levels pre-evening meal and lower mean concentrations before breakfast with detemir compared to NPH.

**Table 3:** Efficacy results after 24 months; ITT

	Detemir		NPH		Detemir—NPH		
				
	n	Mean (SE)	n	Mean (SE)	Mean	(95% CI)	*P*-value
HbA_1c_ (%)	320	7.36 (0.06)	159	7.58 (0.08)	−0.22	(−0.41 to −0.03)	0.022*
FPG_lab_ (mmol/l)	318	8.35 (0.27)	158	9.43 (0.38)	−1.08	(−1.98 to −0.18)	0.019
Weight (kg)	320	72.92 (0.26)	159	73.91 (0.37)	−0.99	(−1.86 to −0.13)	0.024
Hip/waist ratio (cm/cm)	313	1.17 (0.01)	157	1.18 (0.01)	−0.01	(−0.03 to 0.01)	0.277
Abdominal skin-fold thickness (mm)	312	23.48 (0.47)	157	22.54 (0.65)	0.94	(−0.60 to 2.48)	0.231
Leptin (µg/l)	311	11.07 (0.40)	158	11.53 (0.55)	−0.46	(−1.77 to 0.85)	0.493

All end-points were compared between the treatment groups by fitting an analysis of covariance (ANCOVA) with treatment and country as fixed effects and baseline value as a covariate. For hip and waist measurements, an average of the three repeated measurements was taken before calculating the ratio. Mean and standard error (SE) are estimated from the model.

NPH, Neutral Protamine Hagedorn; CI, confidence interval; HbA_1c_, glycated haemoglobin; FPG, fasting plasma glucose.

* The superiority criterion was fulfilled for detemir relative to NPH.

After 24 months, 38% of patients had achieved an HbA_1c_≤ 7.0% with detemir compared to 29% with NPH (*P* = 0.043), while 22% of patients on detemir and 13% on NPH reached this level of control in the absence of confirmed hypoglycaemia during the last month of treatment (*P* = 0.019). Based on SMPG recordings, 52% of patients on detemir and 41% on NPH met the PG target of ≤ 6.0 mmol/l pre-breakfast, while 40% and 32%, respectively, met this target pre-evening meal.

### Hypoglycaemia

Detemir was associated with a 69% lower risk of major hypoglycaemic episodes compared to NPH (*P* < 0.001), while the overall risk of hypoglycaemia was comparable between treatments (Table 4). The highest frequency of hypoglycaemic episodes was observed during the first 3 months of the trial in both treatment groups. The risk of nocturnal hypoglycaemia was 46% lower with detemir than with NPH (*P* < 0.001). The reduced risk of nocturnal episodes was observed regardless of classification and was reflected by a lower number of nocturnal hypoglycaemic episodes per patient per year with detemir relative to NPH (3.4 vs. 6.4 episodes). Nocturnal hypoglycaemia constituted about 13% of all episodes recorded during treatment with detemir compared to 18% with NPH.

**Table 4:** Summary of treatment-emergent hypoglycaemic episodes

	Detemir			NPH			Relative risk (Detemir/NPH)	
			
	Patients *n* (%)	Number of episodes	Episodes/patient/year	Patients *n* (%)	Number of episodes	Episodes/patient/year	Estimate (95% CI)	*P*-value
All	309 (93.4)	15 867	26.2	159 (97.0)	11 052	36.0	0.74 (0.51–1.07)	0.112
Major	49 (14.8)	148	0.2	42 (25.6)	237	0.8	0.31 (0.16–0.58)	< 0.001
Minor	301 (90.9)	13 152	21.7	158 (96.3)	8659	28.2	0.78 (0.52–1.16)	0.220
Symptoms only	221 (66.8)	2334	3.9	122 (74.4)	2026	6.6	0.58 (0.39–0.86)	0.007
Nocturnal	237 (71.6)	2026	3.4	124 (75.6)	1954	6.4	0.54 (0.40–0.71)	< 0.001
Major	18 (5.4)	34	0.1	25 (15.2)	66	0.2	0.27 (0.13–0.57)	0.001
Minor	222 (67.1)	1667	2.8	120 (73.2)	1508	4.9	0.57 (0.42–0.77)	< 0.001
Symptoms only	107 (32.3)	301	0.5	60 (36.6)	358	1.2	0.44 (0.29–0.67)	< 0.001

Three hundred and sixty-three hypoglycaemic episodes could not be classified as major, minor or symptoms only and are only included in the total number of episodes. Several episodes had missing time and were therefore not considered as nocturnal. Forty-six nocturnal hypoglycaemic episodes could not be classified as major, minor or symptoms only. Hypoglycaemic episodes occurring during NPH treatment of patient randomized to detemir are excluded.

NPH, Neutral Protamine Hagedorn; *n*, number of patients having at least one hypoglycaemic episode; %, proportion of patients exposed in the treatment period having an episode; CI, confidence interval.

Hypoglycaemic episodes were reported as serious adverse events for 14 (4.3%) patients on detemir (18 episodes, of which six were comas) compared to 12 (7.3%) patients on NPH (35 episodes, of which eight were comas).

### Weight and body composition

The increase in body weight was less with detemir than with NPH (1.7 kg vs. 2.7 kg; *P* = 0.024). Adjustment for HbA_1c_ at end of trial gave similar results. Slight and similar changes in hip/waist ratio, skin-fold thickness and leptin levels were observed in both groups, but there were no statistically significant differences between groups after 24 months (Table 3).

### Insulin regimen and doses

After 24 months of treatment, the median (sd) daily dose of detemir was 0.56 (0.40) U/kg compared to 0.46 (0.27) IU/kg with NPH and the median doses of aspart were 0.43 (0.29) and 0.38 (0.22) U/kg, respectively. A total of 37% of patients completed the trial on a once-daily detemir regimen compared to 45% on NPH (NS). The median time to transfer from a once-daily to a twice-daily regimen was approximately 9 months with both treatments (NS). In general, the median dose ratio (evening/morning) was 1.2 with detemir compared to 1.5 with NPH.

### Adverse events

Adverse events were reported in about 80% of patients in both groups. These were judged by the investigator as being possibly or probably related to trial drug in 10.9% and 17.1% of all patients exposed to detemir and NPH, respectively. Serious adverse events were reported for about 15–17% of treated patients and were considered possibly or probably related to trial drug in a total of 4.2% and 6.7% of patients treated with detemir and NPH, respectively. The latter difference between treatments could mainly be attributed to a higher frequency of hypoglycaemia and hypoglycaemic coma with NPH.

Four deaths were reported in the detemir group: cardio-respiratory arrest in relation to status epilepticus, sudden death, bronchopneumonia and myocardial infarction following surgery. No deaths were reported in the NPH groups. All of these events were judged as being unlikely related to trial drug by the investigator. Withdrawal because of adverse events was more common with detemir and included eight events considered as possibly or probably related to trial drug. Three of these were non-serious injection site disorders of mild or moderate severity, while the others included hypoglycaemic coma plus humeral fracture, retinal detachment, weight gain and allergic dermatitis.

### Insulin antibodies and other safety data

Antibody formation increased between baseline and 64 weeks of treatment with detemir, but stabilized and tended to decrease during the second year (not shown). Statistical analyses of changes in antibody levels between week 64 and week 105 showed small, but statistically significant, reductions in detemir-specific antibodies (*P* = 0.001), cross-reacting antibodies and aspart-specific antibodies (*P* < 0.05 for both).

No apparent differences were observed between treatments in standard laboratory parameters, vital signs, ECG or fundoscopy/fundus photography during the trial.

## Discussion

This is the first trial to apply a treat-to-target concept with detemir in patients with Type 1 diabetes. This approach was used to achieve and maintain the best possible glycaemic control throughout the trial. After 24 months, mean HbA_1c_ was around 7.4% and 7.6% with detemir and NPH, respectively, reflecting fairly good control even if recommended levels of HbA_1c_ < 7.0%[21] were not achieved. This reflects the fact that optimal glycaemic control is very difficult to attain in a proportion of patients, even with relatively frequent and close contact with the clinical sites, and emphasizes the need for continued focus on insulin dose titration.

Six months into the trial, blinded review of the pre-breakfast and pre-evening meal PG concentrations revealed that PG targets were not achieved in a substantial proportion of patients and a protocol amendment was implemented to ensure more frequent contact between patients and investigators during the last year of the trial. As shown in Fig. 2, the difference in HbA_1c_ between treatments was most pronounced during the last 6 months. This may reflect that full optimization of detemir was only achieved as investigators gained experience and confidence with use of the drug and participants became less afraid of hypoglycaemia. Considering the lag time between effective insulin titration and resulting improvement in HbA_1c_, the most plausible explanation for the lower HbA_1c_ observed with detemir at the end of trial is that it can be titrated more aggressively than NPH without unacceptable risk of hypoglycaemia. Thus, even at a slightly lower mean HbA_1c_, patients treated with detemir had a markedly reduced risk of both major and nocturnal hypoglycaemic episodes while using a higher median basal insulin dose. This is an important finding because hypoglycaemia remains the main barrier for achieving optimal glycaemic control [5,6].

In the DCCT, the event rate for severe hypoglycaemia requiring assistance was 0.61 events per patient per year [22] at a mean HbA_1c_ of 7.2%[23]. In the current trial we find an event rate with detemir of 0.2 episodes per patient per year with detemir and 0.8 with NPH. The DCCT also found the risk of severe hypoglycaemia was greatest during sleep [3]. This is not supported by our study: only 23% (34 of 148) of all major episodes with detemir and 28% (66 of 237) with NPH were nocturnal.

The lower risk of nocturnal hypoglycaemic episodes with detemir is consistent with results from other clinical trials in both Type 1 and Type 2 diabetes [13,15,16,24] and was observed in spite of lower FPG concentrations. This could be related to the reduced within-patient variation in FPG shown for detemir relative to NPH, which is also in line with findings from previous trials [13,14,16,25].

The combination of less within-patient variation in PG [25], longer duration of action and a reduced peak effect observed with detemir relative to NPH [26] probably signify that FPG targets are achieved more easily without additional risk of hypoglycaemia.

Weight gain is a side-effect of insulin therapy, which seems to be inversely correlated to reduction in HbA_1c_[22,27]. Although a gain in weight was also observed with detemir during the trial, the increase was markedly lower than that with NPH in spite of a larger reduction in HbA_1c_. There were no differences in changes in snacking during the trial between the two treatment groups.

Weight gain adversely affects insulin sensitivity, lipid levels and blood pressure and thereby increases the risk of cardiovascular disease [4,7]. Weight gain may have a negative effect on patients’ self-perception and act as a barrier for optimizing insulin [28]. The DCCT showed that patients treated on an intensive insulin regimen had a greater increase in BMI than expected during the 6–9 years of treatment without any tendency to lose accumulated weight [29]. Therefore, limiting the increase in body weight associated with long-term insulin therapy is of major importance.

Treatment with insulin detemir did not give rise to any unexpected safety findings and the adverse event profile did not give reasons for concern. The four deaths reported with detemir were separate events that the investigator considered as having an unlikely relation to trial drug; none of the events were associated with reports of hypoglycaemia.

As expected, administration of detemir gave rise to the formation of insulin antibodies in patients not previously exposed to this insulin preparation. However, antibody levels stabilized and tended to decrease during the second year of treatment even though median doses of detemir increased. Moreover, insulin antibodies did not appear to have any impact on metabolic control.

In conclusion, long-term treatment with detemir using a treat-to-target concept resulted in lower HbA_1c_ levels than NPH with reduced risk of major and nocturnal hypoglycaemia and less weight gain. The general safety profile over 24 months of treatment did not give rise to any concerns or unexpected findings.

## Competing interests

This trial was sponsored by Novo Nordisk A/S, Denmark. The investigators or their institutions were remunerated appropriately for their activity in the study and in some cases for other activities undertaken together with Novo Nordisk. A.P. has received speaking honoraria and institutional research support from Novo-Nordisk, Sanofi-Aventis and Neurocrine Biosciences Inc. P.B. was principal investigator on clinical trials funded by Novo-Nordisk. J.L. is a Novo Nordisk employee and shareholder.

## Abbreviations

ANCOVA: analysis of covariance
DCCT: Diabetes Control and Complications Trial
ECG: electrocardiogram
NPH: Neutral Protamine Hagedorn
NS: not significant
PG: plasma glucose
RIA: radioimmunoassay
SMPG: self-measured plasma glucose
TTT: treat-to-target

## References

[1] The Diabetes Control and Complications Trial Research Group (1993). The effect of intensive treatment of diabetes on the development and progression of long-term complications in insulin-dependent diabetes mellitus. New Engl J Med.

[2] Nathan DM, Cleary PA, Backlund JY, Genuth SM, Lachin JM, Orchard TJ (2005). Intensive diabetes treatment and cardiovascular disease in patients with type 1 diabetes. N Engl J Med.

[3] The Diabetes Control and Complications Research Group (1991). Epidemiology of severe hypoglycemia in the diabetes control and complications trial. Am J Med.

[4] Purnell JQ, Hokanson JE, Marcovina SM, Steffes MW, Cleary PA, Brunzell JD (1998). Effect of excessive weight gain with intensive therapy of type 1 diabetes on lipid levels and blood pressure: results from the DCCT. JAMA.

[5] McCrimmon RJ, Frier BM (1994). Hypoglycaemia, the most feared complication of insulin therapy. Diabete Metab.

[6] Cryer PE (2002). Hypoglycaemia: the limiting factor in the glycaemic management of Type I and Type II diabetes. Diabetologia.

[7] Russell-Jones D, Khan R (2007). Insulin-associated weight gain in diabetes —causes, effects and coping strategies. Diabetes Obes Metab.

[8] Starke AA, Heinemann L, Hohmann A, Berger M (1989). The action profiles of human NPH insulin preparations. Diabetic Med.

[9] Kølendorf K, Bojsen J, Deckert T (1983). Clinical factors influencing the absorption of ^125^I-NPH insulin in diabetic patients. Horm Metab Res.

[10] Owens DR, Zinman B, Bolli GB (2001). Insulins today and beyond. Lancet.

[11] Gough SC (2007). A review of human and analogue insulin trials. Diabetes Res Clin Pract.

[12] Havelund S, Plum A, Ribel U, Jonassen I, Vølund Aa, Markussen J (2004). The mechanism of protraction of insulin detemir, a long-acting, acylated analog of human insulin. Pharm Res.

[13] Russell-Jones D, Simpson R, Hylleberg B, Draeger E, Bolinder J (2004). Effects of QD insulin detemir or neutral protamine Hagedorn on blood glucose control in patients with type I diabetes mellitus using a basal-bolus regimen. Clin Ther.

[14] Home P, Bartley P, Russell-Jones D, Hanaire-Broutin H, Heeg J-E, Abrams P (2004). Insulin detemir offers improved glycemic control compared with NPH insulin in people with type 1 diabetes: a randomized clinical trial. Diabetes Care.

[15] De Leeuw I, Vague P, Selam JL, Peterkova V, Leth G, Gall M-A (2005). Insulin detemir used in basal-bolus therapy in people with type 1 diabetes is associated with a lower risk of nocturnal hypoglycaemia and less weight gain over 12 months in comparison to NPH insulin. Diabetes Obes Metab.

[16] Hermansen K, Fontaine P, Kukolja KK, Raskin P, Boehm BO, Råstam J (2004). Insulin analogues (insulin detemir and insulin aspart) versus traditional human insulins (NPH insulin and regular human insulin) in basal-bolus therapy for patients with type 1 diabetes. Diabetologia.

[17] ICH ICH Harmonised Tripartite Guideline. http://www.ich.org/LOB/media/MEDIA482.pdf.

[18] World Medical Association Declaration of Helsinki: Ethical Principles for Medical Research Involving Human Patients.

[19] Lindholm A, Jensen LB, Home PD, Raskin P, Boehm BO, Råstam J (2002). Immune responses to insulin aspart and biphasic insulin aspart in people with type 1 and type 2 diabetes. Diabetes Care.

[20] Morikawa T, Yoshida M (1995). A useful testing strategy in phase III trials: combined test of superiority and test of equivalence. J Biopharm Stat.

[21] American Diabetes Association (2007). Standards of medical care in diabetes —2007. Diabetes Care.

[22] The Diabetes Control and Complications Trial Research Group (1995). Adverse events and their association with treatment regimens in the diabetes control and complications trial. Diabetes Care.

[23] The Diabetes Control and Complications Trial Research Group (1996). The absence of a glycemic threshold for the development of long-term complications: the perspective of the Diabetes Control and Complications Trial. Diabetes.

[24] Hermansen K, Davies M, Derezinski T, Martinez Ravn G, Clauson P, Home P (2006). A 26-week, randomized, parallel, treat-to-target trial comparing insulin detemir with NPH insulin as add-on therapy to oral glucose-lowering drugs in insulin-naive people with type 2 diabetes. Diabetes Care.

[25] Heise T, Nosek L, Rønn BB, Endal L, Heinemann L, Kapitza C (2004). Lower within-subject variability of insulin detemir in comparison to NPH insulin and insulin glargine in people with type 1 diabetes. Diabetes.

[26] Plank J, Bodenlenz M, Sinner F, Magnes C, Görzer E, Regitting W (2005). A double-blind, randomized, dose–response study investigating the pharmacodynamic and pharmacokinetic properties of the long-acting insulin analog detemir. Diabetes Care.

[27] The Diabetes Control and Complications Trial Research Group (1988). Weight gain associated with intensive therapy in the diabetes control and complications trial. Diabetes Care.

[28] Carver C (2006). Insulin treatment and the problem of weight gain in type 2 diabetes. Diabetes Educ.

[29] The Diabetes Control and Complications Trial Research Group (2001). Influence of intensive diabetes treatment on body weight and composition of adults with type 1 diabetes in the Diabetes Control and Complications Trial. Diabetes Care.

